# Plant Bioactive Compounds in Pre- and Postharvest Management for Aflatoxins Reduction

**DOI:** 10.3389/fmicb.2020.00243

**Published:** 2020-03-12

**Authors:** Martina Loi, Costantino Paciolla, Antonio F. Logrieco, Giuseppina Mulè

**Affiliations:** ^1^Institute of Sciences of Food Production, Italian National Research Council, Bari, Italy; ^2^Department of Biology, University of Bari “Aldo Moro, ” Bari,Italy

**Keywords:** *Aspergillus*, aflatoxins, reduction, bioactive compounds, plant extracts

## Abstract

Aflatoxins (AFs) are secondary metabolites produced by *Aspergillus* spp., known for their hepatotoxic, carcinogenic, and mutagenic activity in humans and animals. AF contamination of staple food commodities is a global concern due to their toxicity and the economic losses they cause. Different strategies have been applied to reduce fungal contamination and AF production. Among them, the use of natural, plant-derived compounds is emerging as a promising strategy to be applied to control both *Aspergillus* spoilage and AF contamination in food and feed commodities in an integrated pre- and postharvest management. In particular, phenols, aldehydes, and terpenes extracted from medicinal plants, spices, or fruits have been studied in depth. They can be easily extracted, they are generally recognized as safe (GRAS), and they are food-grade and act through a wide variety of mechanisms. This review investigated the main compounds with antifungal and anti-aflatoxigenic activity, also elucidating their physiological role and the different modes of action and synergies. Plant bioactive compounds are shown to be effective in modulating *Aspergillus* spp. contamination and AF production both *in vitro* and *in vivo*. Therefore, their application in pre- and postharvest management could represent an important tool to control aflatoxigenic fungi and to reduce AF contamination.

## Introduction

**FIGURE 1:**
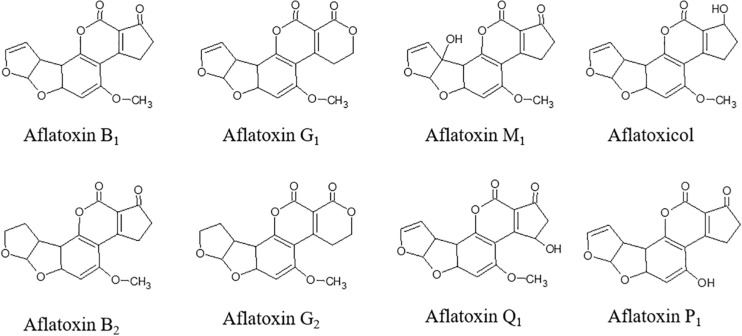
Chemical structure of aflatoxins and their metabolites.

**FIGURE 2:**
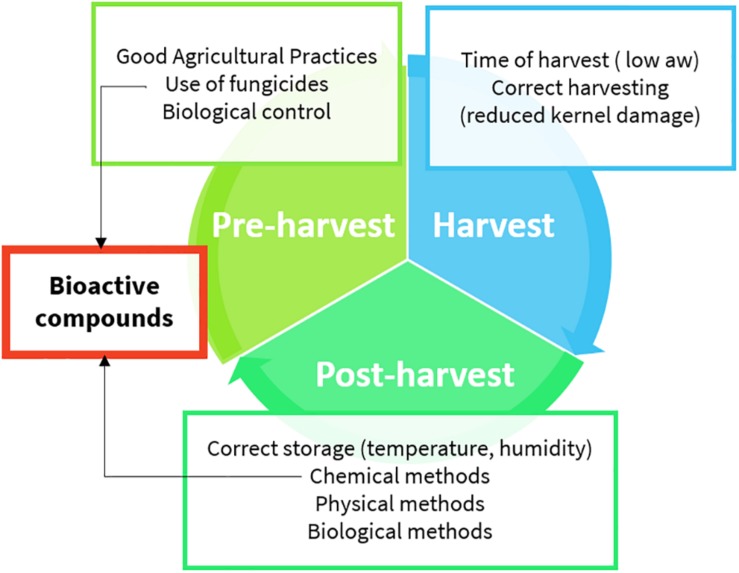
Aflatoxin management practices (details are provided in the text).

Aflatoxins (AFs) are toxic secondary metabolites, mainly produced by *Aspergillus* spp., which are recognized as human carcinogens (AFs of the B and G series) and possible carcinogens (AFs of the M series). They represent a great health concern (Kumar et al., 2017). Toxic outcomes, also known as aflatoxicosis, may include liver cancer, hepatotoxicity, immune system depression, and impaired growth both in humans and animals (IARC, 2012). AF maximum limits are regulated in Europe; products exceeding the maximum levels cannot be placed on the market nor mixed with uncontaminated ones (European Commission, 2006). From a chemical point of view, AFs (Figure 1) are difuranocoumarins composed of two furan rings arranged to a coumarin moiety in a rigid and planar structure (Loi et al., 2017). The high chemical stability endows them with high resistance to heat treatments, extreme pH values, high pressures, and mild (food grade) chemical treatments. As a result, the contamination persists in processed products, including those deriving from animals. Meat, milk, and eggs may also be contaminated with AF metabolites, mainly originating from *in vivo* hydroxylation reactions (AF of the series M, aflatoxicol, aflatoxin Q_1_, and aflatoxin P_1_). AF contamination is a major problem in tropical and subtropical regions, where the environmental conditions are extremely favorable to fungal growth and AF production. However, in the last years, also Mediterranean areas have suffered from severe AF contamination due to climate change, temperature rise, and recurrent droughts (Moretti et al., 2019). AF management is a complex task, requiring actions at every stage of the supply chain (Figure 2). The application of the Good Manufacturing Practices (GAPs), i.e., crop rotation, the use of fungicides, and resistant varieties, is the first critical practice to prevent and reduce fungal contamination. However, the GAPs alone are not sufficient to avoid AF contamination, as it may depend upon several biotic and abiotic factors, also during storage (Mahuku et al., 2019). Therefore, the postharvest management is essential to manage AF contamination throughout the whole supply chain (Leslie and Logrieco, 2014).

*Aspergillus* spp. contamination can be detected in samples by several approaches. A basic microbiological diagnosis with chromogenic substrates was developed for the detection of toxigenic fungi, including *Aspergillus flavus*, *Aspergillus carbonarius*, and *Aspergillus ochraceus*. The great advantage is the use of basic laboratory equipment, a relatively low cost, and time for analysis (48–72 h). However, being a very generic growth test, it can be used only as a rapid screening test (Jefremova et al., 2016). On the contrary, advanced molecular PCR-based tools can be used to tackle conserved genes in *Aspergillus* spp. and AF biosynthetic gene cluster in contaminated materials (Moretti and Susca, 2017).

Controlling humidity, temperature, and moisture are among the most effective management strategies to cope with fungal spoilage and AF production during the storage and transport of susceptible commodities (Neme and Mohammed, 2017). Physical methods, such as sorting, dehulling, cleaning, and milling, are widely used to remove highly contaminated fractions from cereals during processing. Other physical methods include the use of microwave, UV, pulsed light, electrolyzed water, cold plasma, ozone, and irradiation. Despite their potentialities, their use is still limited due to the high technology cost and the residual toxic potential (Mahato et al., 2019).

Biological methods rely on the application of microorganisms (Liuzzi et al., 2017), pure enzymes (Loi et al., 2018), or enzyme extracts (Branà et al., 2020) able to degrade and, possibly, detoxify mycotoxins. In Europe, they can be authorized as postharvest treatments in feed, as long as safety, efficacy, and non-interference with feed nutrients is proved (Commission Regulation (EU) 2015/786,2015).

The use of chemicals to prevent fungal growth in the field, in food, and feed products is a common practice worldwide. The use of fungicides and artificial preservatives has raised concern in consumers, researchers, and stakeholders because of the possible residual toxicity, carcinogenicity, and environmental pollution. The possible development of new resistant fungal strains is also a matter of great concern. Therefore, the use of natural compounds may encounter higher consumers’ and stakeholders’ acceptability (Onaran and Yanar, 2016). Bioactive compounds deriving from plant metabolism belong to greatly diverse chemical groups and possess different biochemical and physiological roles. Therefore, they are considered versatile molecules. Indeed, determining the exact and univocal function of secondary metabolites in plants is a difficult task.

Nonetheless, they share common antimicrobial (Bassolé and Juliani, 2012), antifungal (Tabassum and Vidyasagar, 2013), antioxidant properties (Miguel, 2010), and the capability of improving the postharvest management of vegetable crops (Sivakumar and Bautista-Baños, 2014). Moreover, particular attention is paid to these molecules as bioactive compounds in the human diet because of their high antioxidative capacity (Pisoschi et al., 2016).

Despite their potentialities having been widely investigated in the past, their application as AFs control agents in pre- and postharvest remains still poorly explored. Bioactive compounds have been widely used to inhibit *Aspergillus* growth at different levels (mycelia growth, spore production, germ tube formation), to inhibit the secondary metabolism and AFs production. In addition, their direct use was also found to degrade AFs and, in some cases, detoxify them.

## Bioactive Compounds in Plant Metabolism

Plants are the richest source of bioactive compounds. Bioactive metabolites are classified into primary or secondary metabolites, depending on their functional role (Sharma et al., 2019). Plants and fungi produce thousands of secondary metabolites according to the physiological stage, tissue localization (floral and non-floral leaves, fruits, or bark), environmental conditions, and other biotic or abiotic stress. These compounds may be involved in the primary physiological function of the cell; they may participate in the control of cell growth and cell development, acting as plant growth substances, i.e., plant hormones. Among them, ethylene, auxin, gibberellins, abscisic acid, cytokinins, brassinosteroids, and polyamines are the most important ones (Depuydt et al., 2016). Nevertheless, their main function is ecological, especially with regards to the plant defense against herbivores, bacteria, and fungi (Mithöfer and Maffei, 2017).

Plants cope with pathogen attacks by different types of defense mechanisms, based on either anatomical or biochemical features (passive and constitutive defense), or active changes induced by pathogens (active and inducible defense). In some cases, like for terpenes, compounds can be secreted in low basal amounts constitutively, and expression can be triggered to produce higher amounts upon tissue damage or pathogen attack. Passive or constitutive defense compounds include glucosides, saponins, antifungal proteins, inhibitors of enzymes, and antifeedants, while inducible molecules include phytoalexins, pathogen-related (PR) proteins, chitinases, and glucanases (Walters, 2011).

Metabolites involved in the defense mechanism may occur in glycosylated or conjugated forms, which allow the plant to synthetize and store them in a non-toxic form. The conjugation or their specific localization (i.e., in the vacuoles or other subcellular compartments) are strategies to avoid autoallelopathy and to produce active forms quickly and only when needed (Chaves Lobón et al., 2019).

Conversely, the *de novo* synthesis of antifungal molecules is also observed during the infection process in many plants. These substances are called phytoalexins, and they are similar to the constitutive antifungal toxins, although they show a more lipophilic character. Plants can also produce compounds with animal hormonal activity, the phytoecdysones, which can alter or cause precocious insect development. Finally, they may have a role in establishing the symbiotic processes with beneficial fungi and lichens (Ghasemzadeh and Ghasemzadeh, 2011).

**FIGURE 3:**
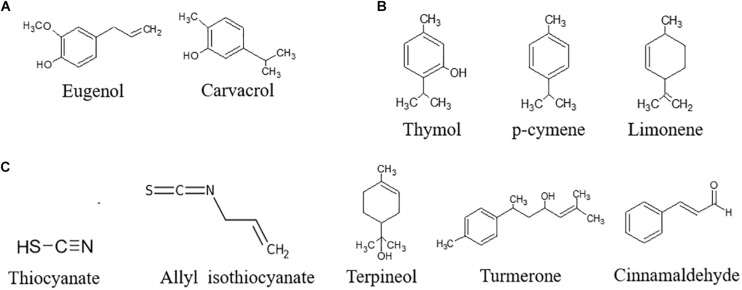
Chemical structure of the most important bioactive secondary compounds with antifungal and antiaflatoxin activity. **(A)** Phenols, **(B)** Terpenes, **(C)** N-containing compounds.

Bioactive compounds can be extracted by different techniques: Soxhlet extraction, maceration, and hydrodistillation are classically used. The use of ultrasounds, microwaves, electric fields, high pressures, or supercritical fluids have been investigated to reduce the use of solvents and apply gentler extraction conditions (Azmir et al., 2013; Giacometti et al., 2018). Water traces can be removed to obtain a concentrated extract, also referred as to essential oil (EO). On the basis of the biosynthetic origin, secondary metabolites can be divided into three main groups: (i) phenolics, (ii) terpenes, and (iii) nitrogen-containing compounds. With regards to the antifungal and antiaflatoxin activity, the most important bioactive secondary compounds are reported in Figure 3.

### (i) Phenolic Compounds

The term phenolic compounds generally includes compounds bearing one or more hydroxylated aromatic rings and are subgrouped into phenolic acids, stilbenes, flavonoids, lignans, and ellargic acids. The flavonoids subgroup comprises a wide variety of simple compounds like anthocyanins, flavonols, chalcones, flavanones, flavones, and isoflavones or complex ones, such as condensed tannins (Zhang and Tsao, 2016). Thanks to their hydroxyl and carboxyl moieties, polyphenols act as antioxidants. They modulate the cellular redox status by directly quenching free radicals and chelating metal ions (promotors of oxidative reactions). They also activate redox-sensitive transcription factors for the antioxidative enzymes (superoxide dismutase, catalase, and glutathione peroxidase) (Upadhyay and Dixit, 2015). Protein binding and inhibition is mediated by hydrogen bonds between hydroxyl moiety of phenols and the carboxyl and thiol groups of proteins. Conversely, the aromatic ring is able to interact with proteins through van der Waals (hydrophobic) interaction.

Structure–activity relationships of two phenol derivatives (cinnamaldehyde and eugenol) were studied on two phytopathogenic fungi, namely, *Rhizoctonia solani* and *Fusarium oxysporum*. Phenol antifungal activity was shown to depend on the chemical structure. In particular, aldehydes, acid groups, conjugated double bonds, and the length of CH chain outside the ring have increased the antifungal activity (Xie et al., 2017). While aldehydes and acid groups may be more prone to react with amino acid residues of proteins through hydrogen bonds and induce conformational modification because of the proton release ability, the length of the CH chain increases hydrophobicity, a major determinant of phenol capability to enter the plasma membrane (Ben Arfa et al., 2006; Dambolena et al., 2011).

### (ii) Terpenes

Terpenes are volatile compounds deriving from the condensation of two or more isoprene molecules. They represent the largest class of plant compounds, with more than 40,000 different chemical structures. They are usually synthetized and stored in trichomes or secretory glands to be secreted constitutively or released as a consequence of tissue damages. Their function in plant metabolism is extremely diverse. Terpenes act as radical scavenging molecules against UV light damage and other environmental stresses. The double bonds can absorb high-energy radiation or scavenge free radicals, functioning as a first defense mechanism. Nonetheless, not all terpenes have a defensive function. Volatile terpenes are generally released constitutively to act as attractants to pollinators and symbionts, repellents to herbivores, or as signaling molecules to other plants or plant tissues. Polyisoprene intermediates are used in the post-translational modification of prenylated proteins (Pichersky and Raguso, 2018). Limonene, carvone, carvacrol, linalool, thymol, terpineol, myrcene, linalool, and pinene are the most important ones, with regards to the antifungal activity against *Aspergillus* spp. The latter activity is mainly due to their lipophilic nature, which allows them to enter the cell and interact with the cellular and mitochondrial membranes, and cause alteration in cell permeability and electrochemical potential (Tian et al., 2012b).

### (iii) Nitrogen-Containing Compounds

Nitrogen-containing compounds are a heterogeneous group, which share the presence of at least one nitrogen atom in their chemical structure: glucosinolates, alkaloids, and cyanogenic glucosides are the main classes. All of them have defensive functions, but only glucosinolates have been recently exploited as antifungal and antiaflatoxin agents (Kaur et al., 2011).

With this regard, volatile compounds from Brussels sprouts (*Brassica oleracea* L. var. gemmifera DC.), cabbage (*Brassica oleracea* L.), kale (*Brassica oleracea* var. sabellica), radish (*Raphanus sativus* L.), and broccoli (*Brassica oleracea* L. var. botrytis L.) were extensively studied. Among them, the most important one is ally-l-isothiocyanate, a β-thioglycoside formed after the hydrolysis of glucosinolates by the enzyme myrosinase (Kumar et al., 2019). Hydrolysis occurs upon tissue damage, since glucosinolates are safely stored in the vacuole. Nitriles may be also produced as secondary products of the reaction. Thiocyanates and nitriles are hydrophilic compounds with high antioxidant capacity. They participate in plant defense systems as allelochemicals, volatile repellents, in the transcriptional regulation of the heat stress response, sulfur metabolism, water transport, stomatal opening, cell growth, and apoptosis (Bones et al., 2015). The isothiocyanate group (–N=C=S) is highly nucleophilic and able to bind thiols, amino groups of amino acids, peptides, and proteins. The antifungal and antiaflatoxin properties are mainly due to the inactivation of crucial enzymes, such as reductases, acetate kinases, and oxidases (Nazareth et al., 2016).

## Antifungal Activity of Bioactive Compounds

Natural plant extracts have been widely used since ancient times for their antimicrobial activity against insects, bacteria, and fungi (Bakkali et al., 2008). Many of them are already employed as pharmaceuticals, feed and food additives, cosmetics and perfume ingredients because of their antioxidant capacity and strong organoleptic properties. Recently, their composition and biological activity have been investigated in relation to the antifungal activity and the ability to inhibit AF production by *Aspergillus* spp.

Carvacrol (Gómez et al., 2018), cinnamaldehyde (Bang et al., 2000; Xie et al., 2004; Tian et al., 2012b; Sun et al., 2016; Khorasani et al., 2017; Gómez et al., 2018), eugenol (Khorasani et al., 2017), limonene (Sharma and Tripathi, 2008; Rammanee and Hongpattarakere, 2011), p-cymene (Pinto et al., 2013), terpineol (Tian et al., 2012b; Kohiyama et al., 2015), thymol (Marei et al., 2012; Gorran et al., 2013; Kohiyama et al., 2015; Shen et al., 2016), and turmerone (Ferreira et al., 2013) are the main active compounds of cinnamon (*Cinnamomum verum* J. Presl), clove [*Syzygium aromaticum* (L.) Merr & L.M. Perry], lemon [*Citrus* × *limon* (L.) Burm. f.], oregano (*Origanum vulgare* L.), and thyme (*Thymus vulgaris* L.) extracts. Their structure is shown in Figure 2, and the main effects as antifungal agents in plant extracts or as pure compounds are presented in Tables 1A and 1B, respectively.

**TABLE 1A:** Antifungal activity of plant extracts on *Aspergillus* spp.

Plant/compounds	Type of extract or oils	Major components	Antifungal activity	Concentration of active compound(s) (mg L^–^^1^ or ml L^–^^1^)	References
Jojoba oil		Gadoleic acid, erucic acid	Growth inhibition of *Aspergillus parasiticus*, *A. ochraceus*, *Fusarium solani*, *Penicillium* sp.	n.p.	Badr et al. (2017)
Jojoba pomace extract	Aqueous isopropyl extract, pH 4–5	84.7% phenols, 15.3% flavonoids			
Jatropha oil		Linoleic acid, oleic acid			
Jatropha pomace extract	Aqueous isopropyl extract, pH 4–5	78.4% phenols, 21.6% flavonoids			
Mentha (*Mentha pulegium L.)*	Aqueous extract	n.p.	Growth inhibition of *A. flavus*	8,000	Omidpanah et al. (2015)
Senna (*Cassia senna* L.)				6000	
Basil (*Ocimum basilicum* L.)				8,000	
Thyme (*Thymus vulgari*s L.)				2,000	
Safflower (*Carthamus tinctorius* L.)				4,000	
Hairy cistus (*Cistus incanus* L.)	Methanolic extract	n.p.	Growth inhibition of *A. parasiticus*; up to 90% of reduction of AFB_1_ production in YES medium and 86% in macadamia nuts	n.p.	Kalli et al. (2018)
Cinnamon (*Cinnamomum zeglanicum* Garcin ex Blume)	Diluted water extract (3, 5, 7, and 9% *v*/*v*)	88.7% cinnamaldehyde	Up to 100% growth inhibition of *Aspergillus flavus* on PDA extract (using 3% extract after 1 day); 100% reduction of AFB_1_ production in pistachio nuts	n.p.	Khorasani et al. (2017)
Clove (*Caryophyllus aromaticus* L.)		71.1% eugenol			
Thyme (*Thymus daenensis* Celak)		Thymol (73.9%) and carvacrol (6.7%)	Up to 100% growth inhibition of *Aspergillus flavus* on PDA extract (using 7% extract after 1 day); up to 100% reduction of AFB_1_ production in pistachio nuts		
Oregano (*Origanum vulgare* L.)	Commercially available essential oil	86% carvacrol	Growth inhibition of *A. parasiticus and A. flavus* in maize extract medium under different environmental conditions (25–37°C, aw 0.99–0.96)	152–505	Gómez et al. (2018)
Cinnamon (*Cinnamomum verum* J. Presl)		66.5% cinnamaldehyde		295–675	
Cinnamon *(Cinnamomum jensenianum* Hand.-Mazz.)	EO obtained by hydrodistillation	17.3% eucalyptol, 12.5% α-terpineol	Growth inhibition of *A. flavus, A. oryzae*, *A. niger*; up to 100% of reduction of AFB_1_ production	2 (*in vitro*)–120 (*in vivo*)	Tian et al. (2012b)
Dill (*Anethum graveolens* L.)	EO obtained by hydrodistillation	n.p.	Growth inhibition of *A. flavus* by disruption of mitochondrial membrane potential (MMP), acidification of external medium, and mitochondrial ATPase and dehydrogenase activities	25–2,000	Tian et al. (2012a)
Dill (*Anethum graveolens* L.)	EO obtained by hydrodistillation	n.p.	100% growth inhibition of *Aspergillus* spp.	2	Tian et al. (2011)
			Up to 86.1 and 94.4% growth inhibition of *Aspergillus* spp. in healthy and wounded cherry tomatoes, respectively	100	
Thyme (*Thymus vulgaris* L.)	EO obtained by hydrodistillation	40.6% borneol, 19.9% α-terpineol, 12.3% camphene	Growth inhibition of *A. flavus*, up to 100% of reduction of AFB_1_ production	1,500 (for AFB_1_ reduction); 2,500 (for *A. flavus* growth inhibition)	Kohiyama et al. (2015)
Thyme (*Thymus daenensis* Celak)	Hydrodistillates resuspended in ethanol	n.p.	Mycelial growth and spore production inhibition of *A. flavus*, up to 100% of reduction of AFB_1_ production	350	Gorran et al. (2013)
Savory (*Satureja khuzestanica*)					
Savory (*Satureja macrosiphonia* Bornm)			Mycelial growth and spore production inhibition of *A. flavus*, up to 56.8% of reduction of AFB_1_ production	500	
Turmeric (*Curcuma longa* L.)	Hydrodistillates resuspended in 5% (*v*/*v*) Tween-20	Oxygenated sesquiterpenes9pc (60.7%) Sesquiterpene hydrocarbons (34.3%)	Up to: 93.41% mycelial growth inhibition; 93.41% spore germination inhibition; 74.6% activity inhibition of mitochondrial ATPase and 84.7% dehydrogenases activity inhibition	n.p.	Hu et al. (2014)
Turmeric (*Curcuma longa* L.)	EO obtained by hydrodistillation	ar-turmerone (33.2%), α-turmerone (23.5%), ß-turmerone (22.7%)	Up to 99.0% inhibition of AFB_1_ production using 5% (*w*/*w*) of extract	n.p.	Ferreira et al. (2013)
Ferula (*Ferulago capillaris Link ex Spreng.)*	EO obtained by hydrodistillation	α-Pinene (35.8%) and limonene (30.9%)	Inhibition of *Aspergillus* spp. growth	640–1,250	Pinto et al. (2013)

*n.p., not provided.*

**TABLE 1B:** Antifungal activity of pure commercial compounds on *Aspergillus* spp.

Plant/compounds	Antifungal activity	Concentration of active compound(s) (mg L^–^^1^ or ml L^–^^1^)	References
Isothiocyanate	Up to 100% of inhibition of *A. parasiticus* growth and aflatoxin production	0.01	Nazareth et al. (2016)
Isothiocyanate	Corn kernels	≥0.00005	Tracz et al. (2017)
Allyl isothiocyanate	Inhibition of *A. flavus* growth and aflatoxin production in corn, barley, and wheat in simulated silo system	0.0005	Quiles et al. (2015)
Allyl isothiocyanate	Inhibition of *Aspergillus parasiticus* growth and aflatoxin production in Brazil nuts	0.0000025	Lopes et al. (2018)
Curcumin	Up to 96.0% inhibition of AFB_1_ production using 0.5% (*w*/*w*) of extract	n.p.	Ferreira et al. (2013)
Cinnamaldehyde	Inhibition of radial growth, spore, and aflatoxin production of *A. flavus*	104	Sun et al. (2016)
Camphene	Mycelial growth inhibition of *F. oxysporum, A niger, P. digitatum;* inhibition of pectin methyl esterase, cellulase, and polyphenol oxidase enzymes	From 121.5 to 314.2	Marei et al. (2012)
(R)-Camphor		From 157.1 to 367.0	
(R)-Carvone		From 432.5 to 120.0	
1,8-Cineole		From 36.4 to 148.4	
Cuminaldehyde		From 79.5 to 363.5	
(S)-Fenchone		From 193.8 to 330.6	
Geraniol		From 73.9 to 357.0	
Carbendazim		From 13.6 to 37.38	
(R)-Linalool		From 266.6 to 73.7	
(1R,2S,5R)-Menthol		From 121.9 to 394.4	
Myrcene		From 95.5 to 336.9	
Thymol		From 20.1 to 50.4	
(S)-Limonene		From 26.8 to 153.2	

*n.p., not provided.*

Those compounds generally act synergistically and in a dose-dependent manner. The highest effects were registered using increasing amounts of bioactive compounds and the whole EOs instead of single compounds (Tian et al., 2012a; Ferreira et al., 2013; Pinto et al., 2013).

**FIGURE 4:**
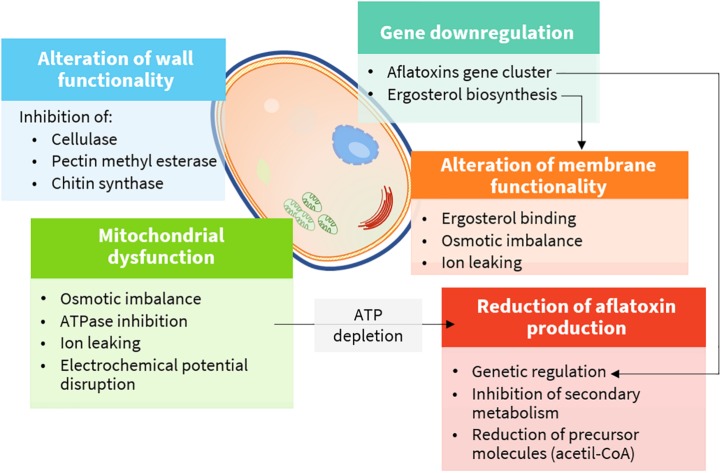
Cellular targets and mechanisms of action of bioactive compounds (details are provided in the text).

**FIGURE 5:**
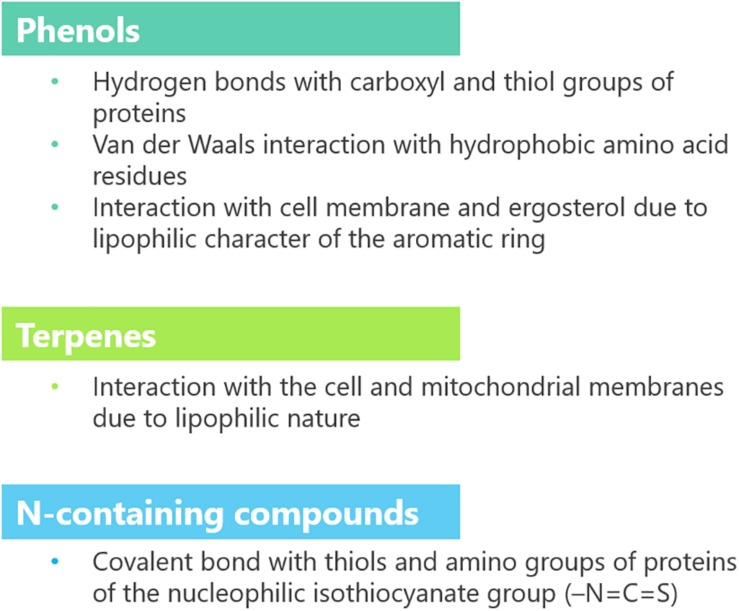
Functional groups and mode of action of bioactive compounds.

Plant extracts are very complex mixtures, and their composition varies according to plant species and chemotype, phenological stage, tissue, and method of extraction (Figueiredo et al., 2008). Accordingly, their effect often has multiple targets (Figure 4) and different modes of action (Figure 5). They induce cytotoxicity through multiple pathways: (i) disrupting cell membrane permeability and functionality; (ii) inhibiting enzymes involved in the synthesis of cell wall components; (iii) impairing ergosterol metabolism; (iv) inducing ultrastructural alterations in cell compartments leading to swelling, vacuolations, and cation leakage; (v) inhibiting cytoplasmic and mitochondrial enzymes; and (vi) altering the osmotic and the redox balance.

### Effects on Cell Wall and Cell Membrane

Fungal cell wall is a dynamic component, essential to assure cell viability. Moreover, it is involved in multiple cell functions, including morphogenesis and pathogenesis. Chitin, glucans, and pectins are the major building blocks, and they are continuously remodeled to cope with cell growth and differentiation by enzymes, such as chitin and glucan synthases, glycohydrolases, and transglycosidases (Gow et al., 2017). Therefore, these enzymes are perfect physiological targets to inhibit fungal growth.

An extensive survey on the antifungal activity of 13 different commercially available monoterpenes was performed by Marei et al. (2012). Among all tested compounds, thymol, followed by limonene, had the highest inhibitory effect on cellulase and pectin methyl esterase enzymes of *Aspergillus niger*, *F*. *oxysporum*, and *Penicillium digitatum*. The rate of inhibition on *A. niger* was higher for the pectin methyl esterase (IC_50_ at 1.28 mg L**^–^**^1^) rather than for the cellulase (IC_50_ at 44.56 mg L**^–^**^1^). Cinnamaldehyde was found to be a non-competitive inhibitor of chitin synthase (IC_50_ at 111.0 mg L**^–^**^1^) and *b*-(1,3)-glucan synthase (IC_50_ at 190.3 mg L**^–^**^1^) (Bang et al., 2000).

Ergosterol is the main sterol derivative of fungi, and it is essential to preserve cell membrane functionality as cholesterol does in animal cells. In addition, it is essential to ensure the activity of membrane-bound enzymes. Owing to its essential role in fungal cells, many fungicides act by inhibiting its biosynthesis or binding it in the cell membrane (Sant et al., 2016). Phenols and aldehydes possess a sufficient hydrophobicity to pass the double phospholipid bilayer, to interact with ergosterol in the cell membrane, or to enter the nucleus and act as regulators for its biosynthesis. As a consequence, alteration of fatty acid profiles along with modification of cell membrane, osmotic imbalance leading to irreversible damage of the hyphae membranes, conidiophores, and death occur (Ansari et al., 2013).

*Cinnamomum* spp. EO or its main component, cinnamaldehyde, were reported to impair ergosterol biosynthesis at concentrations as low as 2 mg L**^–^**^1^ (Tian et al., 2012b) and to cause irreversible deleterious morphological and ultrastructural degenerative alterations of the fungal cell membrane at 104 mg L**^–^**^1^ (Sun et al., 2016; Khorasani et al., 2017). The same effect on fungal morphology was described for *Thymus vulgaris* L. (at 2,500 mg L**^–^**^1^) (Kohiyama et al., 2015), *Curcuma longa* L. (Ferreira et al., 2013; Hu et al., 2014, 2017), and *Anethum graveolens* L. EOs (at 2 and 100 mg L**^–^**^1^ *in vitro* and in cherry tomatoes, respectively) (Tian et al., 2011).

Ergosterol biosynthesis may be regulated at the genomic level. Downregulation of ERG7, ERG11, ERG6, ERG3, and ERG5 genes by citral, the major component of lemongrass EO, was indeed reported for *P. digitatum* (OuYang et al., 2016).

### Mitochondrial Dysfunction

Mitochondrial membrane potential is maintained in healthy cells by an electrochemical gradient through the electron transport chain, which is, ultimately, the major source of ATP molecules. As ATP levels decrease, the normal metabolic functions slow down until cell death occurs. The mechanism of action is not clearly understood. Several hypotheses have been made, including a direct inhibition of ATPases (see *Enzyme Inhibition*) and disruption of the osmotic balance, mainly causing calcium and protons leaking and, consequently, of the electrochemical potential. As for polygodial, a naturally occurring sesquiterpene dialdehyde isolated from different plant species, the mechanism was studied in depth, although with mammalian mitochondrial preparations. In this case, direct inhibition of enzymes was excluded. Indeed, the mechanism was supposed to rely on the uncoupling of the mitochondrial ATPase due to the modification of the electric properties of the membrane surface (Castelli et al., 2005). In yeasts, carvacrol was also responsible for the induction of calcium stress, mediated by the activation of specific intracellular signaling pathways (Rao et al., 2010).

### Enzyme Inhibition

Mitochondrial dysfunction may also occur via ATPase inhibition. Dill (*Anethum graveolens* L.) EO was shown to affect mitochondrial and plasma membrane ATPase at 0.08–0.64 ml L**^–^**^1^ (Pinto et al., 2013), while turmeric (*C. longa* L.) EO was shown to suppress mitochondrial dehydrogenases and mitochondrial ATPase at 2–8 ml L**^–^**^1^ (Hu et al., 2014). Turmeric EO was also found to exert antifungal activity via ATPase, malate dehydrogenase, and succinate dehydrogenase inhibition at 1–8 ml L^–1^ *in vitro* and 4 ml L^–1^ in maize (Hu et al., 2017). The reactivity of phenols and aldehydes in EOs to proteins and enzymes is the major mechanism, as reported for isothiocyanates.

Isothiocyanate were successfully used to inhibit *Aspergillus parasiticus in vitro* at doses of 5 mg (Manyes et al., 2015) or even in gaseous form in foods at concentrations of 100.01 ml L**^–^**^1^ in wheat flour (Nazareth et al., 2016), at ≥0.05 ml L**^–^**^1^ in corn kernels (Tracz et al., 2017), at 0.5 ml L**^–^**^1^ in corn, barley, and wheat in simulated silo system (Quiles et al., 2019), at 0.0025 ml L**^–^**^1^ in Brazil nuts (Lopes et al., 2018), and at 46,040 and 78,250 mg/kg in the Italian “piadina” (Saladino et al., 2016).

## Inhibitory Effect on Aflatoxin B_1_ Production

Aflatoxins are polyketide-derived furanocoumarins, the production of which depends upon 25 different genes, clustered together in a 70-kb DNA sequence region. The majority of the genes encodes for enzymes involved in the synthesis and participates as transcription factors, while others do not have a clear assigned function (Yu et al., 2004).

Many physiological events in fungal cells are regulated by oxidative bursts such as differentiation, switch from conidia to germ tube development, and the onset of secondary metabolism. In particular, oxidants are able to induce AF biosynthesis (Reverberi et al., 2006). In the presence of oxidative stress, the fungal antioxidant molecules (tocopherols, ascorbic acid, carotene, reduced glutathione) and enzymes (superoxide dismutase, catalase, and glutathione peroxidase) are induced concomitantly to AF biosynthetic gene cluster (Reverberi et al., 2010). Therefore, it was also suggested that AF production may also be a way to incorporate oxygen atoms and protect cells from oxidative damage (Kim et al., 2005). The mechanism of EOs or their components may be associated with their antioxidant activity, responsible for the attenuation of the fungal oxidative stress responses, thus AF production (Kim et al., 2005; Reverberi et al., 2005).

Different compounds have been proven to inhibit the production of secondary metabolites like AFB_1_, at comparable or slightly lower concentrations than those that inhibit the mycelial growth, which is consistent with their supposed mode of action. The different inhibition pattern suggests that the suppressive effect is elicited on transcriptional regulators (AflR and AflS) as well as on structural genes (Georgianna and Payne, 2009), as reported in Table 2.

**TABLE 2:** Aflatoxins genes regulated by bioactive compounds.

Gene	Function	Bioactive compound and references
aflC previously known as pksA	Polyketide synthase	Eugenol (Jahanshiri et al., 2015) γ-terpinene (Moon et al., 2018)
aflD previously known as nor-1	Reductase	Eugenol (Jahanshiri et al., 2015; Liang et al., 2015; Lv et al., 2018) Turmeric EO (Hu et al., 2017) γ-terpinene and citral (Moon et al., 2018)
aflE	Reductase	γ-Terpinene (Moon et al., 2018)
aflK	Versicolorin synthase	γ-Terpinene (Moon et al., 2018)
aflL	Desaturase	Citral (Moon et al., 2018)
aflM previously known as ver-1	Dehydrogenase/ketoreductase	Eugenol (Jahanshiri et al., 2015; Liang et al., 2015; Lv et al., 2018) Turmeric EO (Hu et al., 2017)
aflO	Oxidoreductase/P450 monooxygenase	Turmeric EO (Hu et al., 2017) γ-Terpinene and citral (Moon et al., 2018)
aflP previously known as omtA	Methyltransferase	Eugenol (Jahanshiri et al., 2015; Liang et al., 2015; Lv et al., 2018) Turmeric EO (Hu et al., 2017)
aflQ	*O*-Methyltransferase	γ-Terpinene (Moon et al., 2018) Citral (Moon et al., 2018)
aflR	Transcriptional regulator	Eugenol (Jahanshiri et al., 2015 Citral (Moon et al., 2018)
aflS	Transcription enhancer	Citral (Moon et al., 2018)
aflT	Transmembrane protein	Eugenol (Liang et al., 2015; Lv et al., 2018)

Eugenol was proved to be effective in downregulating aflM, aflD, aflC, aflP, aflR (Jahanshiri et al., 2015), aflP, aflM, aflD, and aflT (Liang et al., 2015; Lv et al., 2018) genes. Conversely, turmeric EO downregulated aflD, aflM, aflO, aflP, and aflQ genes (Hu et al., 2017). In a recent study by Moon et al. (2018)γ-terpinene was found to downregulate aflC, aflD, aflE, aflK, aflO, and aflQ genes, whereas citral downregulated aflD, aflE, aflK, aflL, aflO, aflQ, aflR, aflS, aflC, and aflG.

Finally, inhibition of the secondary metabolism as a consequence of the reduced fungal growth and ATP and AF precursor depletion (acetyl coenzyme A) by mitochondrial dysfunction may contribute to the general antiaflatoxigenic effect of these compounds (Tian et al., 2011).

## Aflatoxin Degradation Activity

Various plant extracts were reported to degrade AFB_1_ as well as other mycotoxins both *in vitro* and *in vivo*, as reported in Table 3.

**TABLE 3:** Degradation activity of plant extract on aflatoxin B_1_ (AFB_1_).

Plant	Type of extract/oils	*In vitro*/*in matrix* AFB_1_ reduction	Relative toxicity of AFB_1_ degradation products	References
Araçá (*Psidium cattleianum*)	Aqueous extract	Up to 30% of AFB_1_ degradation (16.67 μg/L) after 48 h of incubation in aqueous medium, pH 6.0–7.0	n.p.	Ponzilacqua et al. (2019)
Rosemary (*Rosmarinus officinalis* L.)		Up to 60% of AFB_1_ degradation (16.67 μg/L) after 48 h of incubation in aqueous medium, pH 6.0–7.0	n.p.	
Oregano (*Origanum vulgare* L.)		Up to 38% of AFB_1_ degradation (16.67 μg/L) after 48 h of incubation in aqueous medium, pH 6.0–7.0	n.p.	
Basil (*Ocimum basilicum* L.)	Aqueous extract	Up to 90% of AFB_1_ degradation (100 μg/L) after 72 h at 60°C in aqueous extract;	70% of mortality reduction by Brine shrimps (*Artemia salina*) bioassay	Iram et al. (2016a)
		*In matrix* degradation (maize) up to 90.4% of degradation after 72 h of incubation at 30°C, pH 8		
Golden tree *(Cassia fistula* L.)		Up to 54% of AFB_1_ degradation (100 μg/L) after 72 h at 60°C in aqueous extract;	n.p.	
		Up to 62.5% of AFB_1_ degradation (100 μg/L, spiked) in maize after 72 h of incubation at 30°C, pH 8		
Ajowan caraway (*Trachyspermum ammi* L.) Sprague ex Turrill	Aqueous extract	Up to 92.8% of AFB_1_ degradation (100 μg/L) after 72 h of incubation at 30°C, pH 8	72% of mortality reduction by Brine shrimps (*Artemia salina*) bioassay	Iram et al. (2016b)
		Up to 89.6% of AFB_1_ degradation (100 μg/L, spiked) in maize after 72 h of incubation at 30°C, pH 8		
Lemon Scented Eucalyptus (*Corymbia citriodora*)	Leaf aqueous extract	Up to 95.21% of AFB_1_ degradation (100 μg/L) after 72 h of incubation at 30°C, pH 8;	75% of mortality reduction by Brine shrimps (*Artemia salina*) bioassay	Iram et al. (2015)
		Up to 70.26% of AFB_1_ degradation (100 μg/L, spiked) in maize after 72 h of incubation at 30°C, pH 8		
Garlic (*Allium sativum* L.)	Aqueous extracts	61.7% of AFB_1_ degradation (50 μg/L) after 1 h of incubation at 37°C in PBS medium; 68.3% after 1 h of incubation at 37°C in real-contaminated sample using 50 mg/L of extract	n.p.	Negera and Washe (2019)
Lemon (*Citrus limon* L.)		56.0% of AFB_1_ degradation (50 μg/g, spiked) after 1 h of incubation at 37°C in PBS medium; 60.6% after 1 h of incubation at 37°C in real-contaminated sample using 50 mg/L of extract		
Thyme (*Thymus daenensis* Celak)	Hydro-distillates	Up to 97% of AFB_1_ degradation (2,000 μg/L) using 2,000 mg/L aqueous extract	n.p.	Gorran et al. (2013)
Savory (Satureja khuzestanica)		Up to 5% of AFB_1_ degradation (2,000 μg/L) using 2,000 mg/L aqueous extract		
Savory (*Satureja macrosiphonia* Bornm)		Up to 13% of AFB_1_ degradation (2,000 μg/L) using 2,000 mg/L aqueous extract		
Ajowan (*Trachyspermum ammi* L.) Sprague ex Turrill	Seeds aqueous extract	Up to 61% of AFB_1_ degradation after incubation at 38°C for 48 h	No chromosomal aberrations induced in corn	Velazhahan et al. (2010)
Basil (*Ocimum tenuiflorum* L.)	Leaves aqueous extract	Up to 74.7% of AFB_1_ degradation after incubation at 85°C for 4 h;	73.7% of cytotoxicity reduction on Hela cells	Panda and Mehta (2013)
		Up to 70.2% of AFB_1_ degradation (1 μg/g, spiked) in rice after 4 h of incubation at 85°C		

*n.p., not provided.*

In most of the studies, the active agents were water soluble, belonged to the flavonoids and phenol groups. Besides the activity of those low molecular weight compounds, the possible coextraction of enzymes able to degrade mycotoxins has to be taken into account. In fact, a detrimental effect on the degrading activity was observed after boiling, while no effect was registered after dialysis with 10–14 kDa cutoff membrane. This suggests that heat-sensitive, high-molecular weight compounds may play a significant role in AF degradation (Vijayanandraj et al., 2014; Ponzilacqua et al., 2019). Indeed, many enzymes, also belonging to plants, have been described for their ability to degrade AFs (Loi et al., 2017; Lyagin and Efremenko, 2019). Among them, polyphenol oxidases and laccases may also use low molecular weight compounds as redox mediators, thus enhancing their degradation capability through a synergistic or additive mechanism (Loi et al., 2018).

Although the mechanism of action is not clearly understood, some authors evaluated the outcome of the degradation by high-performance liquid chromatography (HPLC) and liquid chromatography tandem MS (LC-MS/MS). The chemical properties of AFs were deeply modified upon incubation with plant extracts. AFB_1_ was modified in different ways, including the removal of the double bond of the furan ring and the modification of the lactone ring, resulting in a significant decrease in the cytotoxicity, evaluated on Hela cells (Velazhahan et al., 2010) and by Brine shrimps (*Artemia salina*) bioassay (Iram et al., 2015, 2016a,b). The toxic and carcinogenic potential of AFB_1_ was indeed attributed to the difuran ring, which *in vivo* is quickly oxidized to 8,9-epoxy-AFB_1_ and, to a lesser extent, to the lactone moiety (Loi et al., 2016).

## Discussion

The use of natural compounds in pre- and postharvest appears appealing, especially when compared to the use of antibiotics or fungicides from synthetic origin. Natural flavoring compounds derived from plants were listed as GRAS compounds in Europe and the United States: among others, clove, marjoram, thyme, nutmeg, basil, mustard, and cinnamon. However, despite their proven *in vitro* efficacy and their GRAS status, the use of those compounds as a pre- or postharvest treatment has different limitations: high volatility, poor stability due to oxidation reactions, and strong organoleptic features. This latter may lead to unpleasant tastes and off-flavors in food and feed or interfere with the signaling pathway mechanisms mediated by volatile compounds in the field. To overcome these limitations, different technologies have been studied to deliver bioactive components while preserving them from unwanted chemical reactions and controlling the organoleptic impact. Emulsification, spray drying, coaxial electrospray system, freeze drying, coacervation, *in situ* polymerization, extrusion, fluidized bed coating, and supercritical fluid technology are the most promising ones (Bakry et al., 2016). EOs can be also incorporated in edible coatings (Peretto et al., 2014; Alotaibi et al., 2019), films (Giteru et al., 2015), or even sprayed on food in a vapor form (Gao et al., 2014).

Among the different proposed technologies, the encapsulation of EOs has many advantages, i.e., even dispersion and release of EOs, odor masking, increased shelf life, and improved technological properties (easy dosing and pouring, increased solubility, dust-free material) (Wu et al., 2012; da Rosa et al., 2015).

The antifungal activity of encapsulated eugenol, menthol, and t-anethole (Kumar et al., 2019), *Illicium verum* Hook. f. (Dwivedy et al., 2018), *Cinnamomum zeylanicum* Garcin ex Blume (Kiran et al., 2016), and *Coriandrum sativum* L. (Das et al., 2019). EOs was investigated *in vitro* toward *A. flavus*, and was shown to reduce AFB_1_ production with promising results. A recent study by Mateo et al. (2017) investigated the antiaflatoxigenic potential of a bioactive packaging based on ethylene-vinyl alcohol copolymer films incorporating EOs from *O. vulgare* L., *C. zeylanicum* Garcin ex Blume, or their major active constituents, carvacrol and cinnamaldehyde. On the contrary, the antifungal activity of allyl isothiocyanate was completely lost upon encapsulation (Janatova et al., 2015). This means that specific delivery systems have to be developed for each EO or bioactive compound.

Moreover, the effectiveness of the preharvest treatments also depends upon several biotic and abiotic factors. The treatment response may vary according to the specific plant species or cultivar, due to the activation of cultivar-specific defense pathways and different host–pathogen interaction patterns (Feliziani et al., 2015). Weather conditions and the phenological stage at the delivery may also affect the results of the treatment in the field.

Few *in vivo* trials were conducted to evaluate the efficacy of the use of natural compounds as antifungal agents, even though they focused on the reduction in the postharvest decay (Sivakumar and Bautista-Baños, 2014; Feliziani et al., 2015).

As regards the postharvest treatments, food matrix and composition, lipid content, water activity, pH, and enzymes can decrease their effectiveness as an antimicrobial or antifungal compound (Hyldgaard et al., 2012). Therefore, with respect to the *in vitro* studies, 1–3% higher amounts may be needed to achieve the same results (Firouzi et al., 2007). Nonetheless, when high amounts are used, the organoleptic properties of the food may be impaired. To overcome this issue, lower concentrations with bacteriostatic or fungistatic effects can be used, or they can be applied in combination with other antimicrobial compounds in a “multiple-hurdle approach” (Prakash et al., 2015; Sudharsan et al., 2019). Few authors evaluated the application in food to reduce AFB_1_ contamination, mainly nuts like macadamia (Kalli et al., 2018) and pistachio (Khorasani et al., 2017), obtaining comparable results with respect to the *in vitro* analyses.

### Feed Applications

Bioactive compounds are used in feed to enhance (i) the organoleptic characteristics of feed (as feed flavorings), (ii) feed stability (as antioxidants), and (iii) feed digestibility and gut flora stability (as zootechnical additives) [Regulation (EC) No 1831/2003,2003].

The European Commission approved the use of linalool, thymol, eugenol, carvone, cinnamaldehyde, vanillin, carvacrol, citral, and limonene as flavorings in food products with no restriction. A stepwise approach was adopted to evaluate the safety of those compounds, including the evaluation of the structure–activity relationships, intake from current uses, toxicological threshold of concern, and available data on metabolism and toxicity [Commission Implementing Regulation (EU) No 872/2012,2012].

Simple and substituted phenols like thymol and carvacrol, have been proposed so far as flavoring additives in feed for all animal species; thus, the demonstration of efficacy was not considered necessary for their approval by the European Food Safety Authority [EFSA], 2012. Thanks to their antioxidant capacity, these compounds enhance the stability, the quality, the palatability of animal feed, and prolong the shelf life.

The so-called “phytogenic” feed additives (PFAs) are simple or complex mixtures of compounds belonging to a wide variety of herbs, spices, EO, or non-volatile extracts, which can be used in feed for various purposes. PFAs can be applied as solid powders, granulated, or also in liquid form to premixtures or complete feeds (Steiner and Syed, 2015).

Bioactive compounds are widely used as zootechnical additives to increase animals weight gain and performance. A general positive effect was shown for feed intake, weight gain, and feed conversion rate in piglets, sows, and poultry, while inconsistent data were registered for apparent digestibility in piglets (Franz et al., 2010; Christaki et al., 2012; Zeng et al., 2015) possibly due to improved secretion of digestive enzymes and bile secretion (Hafeez et al., 2015). A positive effect on gut microbiota in monogastric animals was also reported by several authors (Tiihonen et al., 2010; Bento et al., 2013). On the contrary, there is still no evidence of the *in vivo* efficacy on ruminants, while discordant data are available from *in vitro* studies with ruminal models. EOs may improve nitrogen uptake and energy production but at the same time be toxic for the ruminal microbiota, which produces volatile fatty acid and inhibits ruminal methanogenesis (Giannenas et al., 2013).

Two feed additives made of a mixture of encapsulated EOs (carvacrol, methyl salicylate and L-menthol, thymol, D-carvone) from oregano (*O. vulgare* L.) and from caraway seed (*Carum carvi* L.) were positively evaluated by EFSA as growth enhancers for weaned piglets, chickens for fattening, chickens reared for laying, and minor avian species to the point of lay (European Food Safety Authority [EFSA], 2019a, b).

Despite the different uses in animal nutrition, the use as AF-reducing agents in feed is still unexplored. To be used as a feed additive to reduce AF contamination, EOs shall undergo a scientific assessment by EFSA to assure that several requirements are met: (i) the chemical compound is fully characterized and safe to be used; (ii) it leads to an irreversible and effective detoxification; (iii) the products of the detoxification process are not harmful or are less harmful than the contaminant itself to animals, people, or the environment; and (iv) the chemical and organoleptic characteristics of the feed are not altered (Commission Regulation (EU) 2015/786,2015). A clear gap of knowledge for the identification of the degradation products and the evaluation of their toxicity currently limits this application.

*In vivo* studies often show low reliability because the EO composition is usually not fully characterized and active compounds quantified; the effects are not clearly defined because there may be differences in gastrointestinal tract anatomy and functionality also within the same species. When the studies are commercially oriented, some information may be voluntarily scarce (Stevanović et al., 2018). Eventually, limited information is available regarding the interaction between EOs and feed ingredients or other feed additives, such as fibers, probiotics, vitamins, and organic acids (Zeng et al., 2015).

## Conclusion and Future Perspectives

Bioactive compounds from plant species are recognized for their pharmacological and nutraceutical value and are endowed with antifungal and antiaflatoxin activities.

The application of natural compounds deriving from plants to control aflatoxigenic fungi and AF production has been explored mostly *in vitro* in the last 10 years. The mechanisms of action are diverse and mainly target the cell wall, the plasmatic membrane, proteins, and the mitochondrial functionality of fungal cells. Some compounds also act as downregulators of AF biosynthetic pathway, while others have a direct degrading activity toward AF molecules. Limited studies evaluate the applicability of such compounds in food and feed to reduce *Aspergillus* spp. and AFs contamination. Nonetheless, many compounds possess the GRAS status and can be used as food and feed additives in Europe. Bioactive compounds are used as flavoring, antioxidant, and zootechnical additives to improve weight gain and digestibility of feeds in non-ruminant species. Exploring new technologies to extract and use antifungal compounds from food wastes, such as olive oil wastewater or winery by-products, or to deliver such compounds can increase sustainability and lower the cost of these compounds.

Enriching and expanding the genetic repertoire of plant secondary metabolites could help in increasing the plant defense systems. The identification of biosynthetic pathways, plant–host interactions, and varieties with higher content of bioactive compounds are crucial to allow the production of molecules of high commercial value and to improve the safety and quality of plant products. Another possible strategy to counteract AF contamination may be to increase the production of bioactive compound in susceptible commodities.

The major challenges that have to be overcome are the characterization of the active(s) compounds, the standardization of doses and biological activity, the evaluation of interactions in the field or with the food/feed matrix, the identification and the toxicological characterization of the degradation products in the case of the application to AF-contaminated commodities. Nonetheless, the potentialities of these compounds are diverse and may represent a powerful to counteract *Aspergillus* spp. contamination and AF production both in pre- and postharvest.

## Author Contributions

ML and CP wrote the manuscript. AL conceived the review. GM coordinated the contributions. All authors contributed to manuscript revision, read and approved the submitted version.

## Conflict of Interest

The authors declare that the research was conducted in the absence of any commercial or financial relationships that could be construed as a potential conflict of interest.

## References

[B1] Alotaibi M. A. Tayel A. A. Zidan N. S. El Rabey H. A. (2019). Bioactive coatings from nano–biopolymers/plant extract composites for complete protection from mycotoxigenic fungi in dates *J. Sci. Food Agric.* 99 4338–4343. 10.1002/jsfa.9667 30828809

[B2] Ansari M. A. Anurag A. Fatima Z. Hameed S. (2013). “Natural phenolic compounds: a potential antifungal agent,” in *Microbial Pathogens and Strategies for Combating Them: Science, Technology and Education*, ed. Méndez-Vilas A., (Badajoz: Formatex Research Center), 189–195.

[B3] Azmir J. Zaidul I. S. M. Rahman M. M. Sharif K. M. Mohamed A. Sahena F. (2013). Techniques for extraction of bioactive compounds from plant materials: a review. *J. Food Eng.* 117 426–436. 10.1016/j.jfoodeng.2013.01.014

[B4] Badr A. N. Shehata M. G. Abdel-Razek A. G. (2017). Antioxidant activities and potential impacts to reduce aflatoxins utilizing jojoba and jatropha oils and extracts. *Int. J. Pharmacol.* 13 1103–1114. 10.3923/ijp.2017.1103.1114

[B5] Bakkali F. Averbeck S. Averbeck D. Idaomar M. (2008). Biological effects of essential oils–a review. *Food Chem. Toxicol.* 46 446–475. 10.1016/j.fct.2007.09.106 17996351

[B6] Bakry A. M. Abbas S. Ali B. Majeed H. Abouelwafa M. Y. Ahmed M. (2016). Microencapsulation of oils: a comprehensive review of benefits, techniques, and applications. *Compr. Rev. Food Sci. Food Saf.* 15 143–182. 10.1111/1541-4337.12179 33371581

[B7] Bang K. H. Lee D. W. Park H. M. Rhee Y. H. (2000). Inhibition of fungal cell wall synthesizing enzymes by trans-cinnamaldehyde. *Biosci. Biotechnol. Biochem.* 64 1061–1063. 10.1271/bbb.64.1061 10879482

[B8] Bassolé I. H. N. Juliani H. R. (2012). Essential oils in combination and their antimicrobial properties. *Molecules* 17 3989–4006. 10.3390/molecules17043989 22469594 PMC6268925

[B9] Ben Arfa A. Combes S. Preziosi-Belloy L. Gontard N. Chalier P. (2006). Antimicrobial activity of carvacrol related to its chemical structure. *Lett. Appl. Microbiol* 43 149–154. 10.1111/j.1472-765X.2006.01938.x 16869897

[B10] Bento M. H. L. Ouwehand A. C. Tiihonen K. Lahtinen S. Nurminen P. Saarinen M. (2013). Essential oils and their use in animal feeds for monogastric animals - effects on feed quality, gut microbiota, growth performance and food safety: a review. *Vet. Med.* 58 449–458. 10.17221/7029-VETMED

[B11] Bones A. M. Hara M. Rossiter J. T. Kissen R. (2015). Physiology and cellular mechanisms of isothiocyanates and other glucosinolate degradation products in plants. *Front. Plant Sci.* 6:1105. 10.3389/fpls.2015.01105 26697052 PMC4673302

[B12] Branà M. T. Sergio L. Haidukowski M. Logrieco A. F. Altomare C. (2020). Degradation of Aflatoxin B1 by a sustainable enzymatic extract from spent mushroom substrate of *Pleurotus eryngii*. *Toxins* 12:E49. 10.3390/toxins12010049 31947703 PMC7020430

[B13] Castelli M. V. Lodeyro A. F. Malheiros A. Zacchino S. A. Roveri O. A. (2005). Inhibition of the mitochondrial ATP synthesis by polygodial, a naturally occurring dialdehyde unsaturated sesquiterpene. *Biochem. Pharmacol.* 70 82–89. 10.1016/j.bcp.2005.04.016 15894293

[B14] Chaves Lobón N. Ferrer de la Cruz I. Alías Gallego J. C. (2019). Autotoxicity of diterpenes present in leaves of *Cistus ladanifer* L. *Plants* 8:27. 10.3390/plants8020027 30678267 PMC6410244

[B15] Christaki E. Bonos E. Giannenas I. Florou-Paneri P. (2012). Aromatic plants as a source of bioactive compounds. *Agriculture* 2 228–243. 10.3390/agriculture2030228

[B16] Commission Implementing Regulation (EU) No 872/2012 (2012). *Commission Implementing Regulation (EU) No 872/2012 The list of Flavouring Substances Provided for by Regulation (EC) No* 2232/*96 of the European Parliament and of the Council, introducing it in Annex I to Regulation (EC) No 1334/2008 of the European Parliament and of the Council and repealing Commission Regulation (EC) No 1565/2000 and Commission Decision 1999/217/EC.* Available at: http://data.europa.eu/eli/reg_impl/2012/872/oj (accessed October 1, 2012).

[B18] Commission Regulation (EU) 2015/786 (2015). *Commission Regulation (EU) 2015/786 Defining Acceptability Criteria for Detoxification Processes Applied to Products Intended for Animal Feed as Provided for in Directive 2002/32/EC of the European Parliament and of the Council.* Available at: http://data.europa.eu/eli/reg/2015/786/oj (accessed May 19, 2015).

[B19] da Rosa C. G. Maciel M. V. D. O. B. de Carvalho S. M. de Melo A. P. Z. Jummes B. da Silva T. (2015). Characterization and evaluation of physicochemical and antimicrobial properties of zein nanoparticles loaded with phenolics monoterpenes. *Colloids Surf. Physicochem. Eng. Aspects* 481 337–344. 10.1016/j.colsurfa.2015.05.019

[B20] Dambolena J. S. Zygadlo J. A. Rubinstein H. R. (2011). Antifumonisin activity of natural phenolic compounds: a structure–property–activity relationship study. *ý Int. J. Food Microbiol.* 145 140–146. 10.1016/j.ijfoodmicro.2010.12.001 21195498

[B21] Das S. Singh V. K. Dwivedy A. K. Chaudhari A. K. Upadhyay N. Singh P. (2019). Encapsulation in chitosan-based nanomatrix as an efficient green technology to boost the antimicrobial, antioxidant and in situ efficacy of *Coriandrum sativum* essential oil. *Int. J. Biol. Macromol.* 133 294–305. 10.1016/j.ijbiomac.2019.04.070 30986458

[B22] Depuydt S. Van Praet S. Nelissen H. Vanholme B. Vereecke D. (2016). “How plant hormones and their interactions affect cell growth,” in *Molecular Cell Biology of the Growth and Differentiation of Plant Cells*, ed. Rose R. J., (Boca Raton, FL: CRC Press), 174–195. 10.1201/b20316-14

[B23] Dwivedy A. K. Singh V. K. Prakash B. Dubey N. K. (2018). Nanoencapsulated *Illicium verum* Hook. f. essential oil as an effective novel plant-based preservative against aflatoxin B1 production and free radical generation. *Food Chem. Toxicol.* 111 102–113. 10.1016/j.fct.2017.11.007 29126800

[B24] European Commission (2006). *Commission Regulation (EC) No 1881/2006 of 19 December 2006 Setting Maxirtain Contaminants in Foodstuffs.* Available at: http://data.europa.eu/eli/reg/2006/1881/oj (accessed September 12, 2019).

[B25] European Food Safety Authority [EFSA] (2012). Scientific Opinion on the safety and efficacy of phenol derivatives containing ring-alkyl, ring-alkoxy and side-chains with an oxygenated functional group (chemical group 25) when used as flavourings for all species. *Panel Addit. Prod. Substan. Anim. Feed* 10 2573 10.2903/j.efsa.2012.2573

[B26] European Food Safety Authority [EFSA] (2019a). FEEDAP Panel (EFSA Panel on Additives and Products or Substances used in Animal Feed) Bampidis, V., Azimonti, G., Bastos, M.L., Christensen, H., Dusemund, B., Kouba, M., Kos Durjava. M., Lopez-Alonso, M., Lopez Puente, S., Marcon, F., Mayo, B., Pechova, A., Petkova, M., Ramos, F., Sanz, Y., Villa. R, E, Woutersen. R, Chesson, A., Gropp, J., Martelli, G., Renshaw, D., Lopez-Galvez, G.,. Mantovani, A. 2019. Scientific Opinion on the safety and efficacy of Biomin^®^ DC-C as a zootechnical feed additive forweaned piglets. *EFSA Journal* 17 5688 10.2903/j.efsa.2019.5688

[B27] European Food Safety Authority [EFSA] (2019b). FEEDAP Panel (EFSA Panel on Additives and Products or Substances used in Animal Feed), Bampidis, V., Azimonti, G., Bastos, M.L., Christensen, H., Dusemund, B., Kouba, M., Kos Durjava. M., Lopez-Alonso, M., Lopez Puente, S., Marcon, F., Mayo, B., Pechova, A., Petkova, M., Ramos, F., Sanz, Y., Villa. R, E, Woutersen. R, Chesson, A., Gropp, J., Martelli, G., Renshaw, D., Lopez-Galvez, G. Mantovani, A. 2019. Scientific Opinion on the safety and efficacy of Biomin^®^ DC-P as a zootechnical feed additive for chickens for fattening, chickens reared for laying and minor avian species to the point of lay. *EFSA J.* 17:5724 10.2903/j.efsa.2019.5724

[B28] Feliziani E. Landi L. Romanazzi G. (2015). Preharvest treatments with chitosan and other alternatives to conventional fungicides to control postharvest decay of strawberry. *Carbohyd. Polym.* 132 111–117. 10.1016/j.carbpol.2015.05.078 26256331

[B29] Ferreira F. D. Kemmelmeier C. Arrotéia C. C. da Costa C. L. Mallmann C. A. Janeiro V. (2013). Inhibitory effect of the essential oil of *Curcuma longa* L. and curcumin on aflatoxin production by *Aspergillus flavus* Link. *Food Chem.* 136 789–793. 10.1016/j.foodchem.2012.08.003 23122128

[B30] Figueiredo A. C. Barroso J. G. Pedro L. G. Scheffer J. J. C. (2008). Factors affecting secondary metabolite production in plants: volatile components and essential oils. *Flavour Fragr. J.* 23 213–226. 10.1002/ffj.1875

[B31] Firouzi R. Shekarforoush S. S. Nazer A. H. K. Borumand Z. Jooyandeh A. R. (2007). Effects of essential oils of oregano and nutmeg on growth and survival of *Yersinia enterocolitica* and *Listeria monocytogenes* in barbecued chicken. *J. Food Prot.* 70 2626–2630. 10.4315/0362-028X-70.11.2626 18044446

[B32] Franz C. Baser K. H. C. Windisch W. (2010). Essential oils and aromatic plants in animal feeding–a European perspective. A review. *Flavour Fragr. J.* 25 327–340. 10.1002/ffj.1967

[B33] Gao M. Feng L. Jiang T. (2014). Browning inhibition and quality preservation of button mushroom (*Agaricus bisporus*) by essential oils fumigation treatment. *Food Chem.* 149 107–113. 10.1016/j.foodchem.2013.10.073 24295683

[B34] Georgianna D. R. Payne G. A. (2009). Genetic regulation of aflatoxin biosynthesis: from gene to genome. *Fungal Genet. Biol.* 46 113–125. 10.1016/j.fgb.2008.10.011 19010433

[B35] Ghasemzadeh A. Ghasemzadeh N. (2011). Flavonoids and phenolic acids: role and biochemical activity in plants and human. *J. Med. Plant Res.* 5 6697–6703. 10.5897/JMPR11.1404 27399664

[B36] Giacometti J. Kovaèević D. B. Putnik P. Gabrić D. Bilušić T. Krešić G. (2018). Extraction of bioactive compounds and essential oils from Mediterranean herbs by conventional and green innovative techniques: a review. *Food Res. Int.* 113 245–262. 10.1016/j.foodres.2018.06.036 30195519

[B37] Giannenas I. Bonos E. Christaki E. Florou-Paneri P. (2013). Essential oils and their applications in animal nutrition. *Med. Aromat. Plants* 2:140 10.4172/2167-0412.1000140

[B38] Giteru S. G. Coorey R. Bertolatti D. Watkin E. Johnson S. Fang Z. (2015). Physicochemical and antimicrobial properties of citral and quercetin incorporated kafirin-based bioactive films. *Food Chem.* 168 341–347. 10.1016/j.foodchem.2014.07.077 25172719

[B39] Gómez J. V. Tarazona A. Mateo-Castro R. Gimeno-Adelantado J. V. Jiménez M. Mateo E. M. (2018). Selected plant essential oils and their main active components, a promising approach to inhibit aflatoxigenic fungi and aflatoxin production in food. *Food Addit. Contam. A* 35 1581–1595. 10.1080/19440049.2017.1419287 29338637

[B40] Gorran A. Farzaneh M. Shivazad M. Rezaeian M. Ghassempour A. (2013). Aflatoxin B1-reduction of *Aspergillus flavus* by three medicinal plants (Lamiaceae). *Food Control* 31 218–223. 10.1016/j.foodcont.2012.09.024

[B41] Gow N. A. R. Latge J. P. Munro C. A. (2017). The fungal cell wall: structure, biosynthesis, and function. *Microbiol. Spectr.* 5:FUNK-0035-2016. 10.1128/microbiolspec.FUNK-0035-2016. 28513415 PMC11687499

[B42] Hafeez A. Männer K. Schieder C. Zentek J. (2015). Effect of supplementation of phytogenic feed additives (powdered vs. encapsulated) on performance and nutrient digestibility in broiler chickens. *Poult. Sci.* 95 622–629. 10.3382/ps/pev368 26706360

[B43] Hu Y. Kong W. Yang X. Xie L. Wen J. Yang M. (2014). GC–MS combined with chemometric techniques for the quality control and original discrimination of *Curcumae longae* rhizome: analysis of essential oils. *J. Sep. Sci.* 37 404–411. 10.1002/jssc.201301102 24311554

[B44] Hu Y. Zhang J. Kong W. Zhao G. Yang M. (2017). Mechanisms of antifungal and anti-aflatoxigenic properties of essential oil derived from turmeric (*Curcuma longa* L.) *on Aspergillus flavus*. *Food Chem.* 220 1–8. 10.1016/j.foodchem.2016.09.179 27855875

[B45] Hyldgaard M. Mygind T. Meyer R. L. (2012). Essential oils in food preservation: mode of action, synergies, and interactions with food matrix components. *Front. Microbiol.* 3:12. 10.3389/fmicb.2012.00012 22291693 PMC3265747

[B46] IARC. (2012). “Aflatoxins. Monographs on the Evaluation of Carcinogenic Risks to Humans. Chemical agents and related occupations: a review of human carcinogens,” in *Proceedings of the International Agency for the Research on Cancer*, Vol. 100F Lyon, 225–244.

[B47] Iram W. Anjum T. Iqbal M. Ghaffar A. Abbas M. (2015). Mass spectrometric identification and toxicity assessment of degraded products of aflatoxin B1 and B2 by *Corymbia citriodora* aqueous extracts. *Sci. Rep.* 5:14672. 10.1038/srep14672 26423838 PMC4589780

[B48] Iram W. Anjum T. Iqbal M. Ghaffar A. Abbas M. (2016a). Structural elucidation and toxicity assessment of degraded products of aflatoxin B1 and B2 by aqueous extracts of *Trachyspermum ammi*. *Front. Microbiol.* 7:346. 10.3389/fmicb.2016.00346 27064492 PMC4811950

[B49] Iram W. Anjum T. Iqbal M. Ghaffar A. Abbas M. Khan A. M. (2016b). Structural analysis and biological toxicity of aflatoxins B1 and B2 degradation products following detoxification by *Ocimum basilicum* and *Cassia fistula* aqueous extracts. *Front. Microbial.* 7:1105. 10.3389/fmicb.2016.01105 27471501 PMC4943962

[B50] Jahanshiri Z. Shams-Ghahfarokhi M. Allameh A. Razzaghi-Abyaneh M. (2015). Inhibitory effect of eugenol on aflatoxin B1 production in *Aspergillus parasiticus* by downregulating the expression of major genes in the toxin biosynthetic pathway. *World J. Microbiol. Biotechnol.* 31 1071–1078. 10.1007/s11274-015-1857-7 25896772

[B51] Janatova A. Bernardos A. Smid J. Frankova A. Lhotka M. Kourimská L. (2015). Long-term antifungal activity of volatile essential oil components released from mesoporous silica materials. *Ind. Crops Prod.* 67 216–220. 10.1016/j.indcrop.2015.01.019

[B52] Jefremova M. Ostrý V. Malíø F. Ruprich J. (2016). Rapid diagnostic testing of toxigenic microfungi isolated from foodstuffs. *Acta Vet. Brno* 85 151–156. 10.2754/avb201685020151

[B53] Kalli V. Kollia E. Roidaki A. Proestos C. Markaki P. (2018). *Cistus incanus* L. *extract inhibits aflatoxin B*1 production by *Aspergillus parasiticus* in macadamia nuts. *Ind. Crops Prod.* 111 63–68. 10.1016/j.indcrop.2017.10.003

[B54] Kaur R. Rampal G. Vig A. P. (2011). Evaluation of antifungal and antioxidative potential of hydrolytic products of glucosinolates from some members of Brassicaceae family. *J. Plant Breed. Crop Sci*. 3 218–228.

[B55] Khorasani S. Azizi M. H. Barzegar M. Hamidi-Esfahani Z. Kalbasi-Ashtari A. (2017). Inhibitory effects of cinnamon, clove and celak extracts on growth of *Aspergillus flavus* and its aflatoxins after spraying on pistachio nuts before cold storage. *J. Food Saf.* 37:e12383 10.1111/jfs.12383

[B56] Kim J. H. Campbell B. C. Yu J. Mahoney N. Chan K. L. Molyneux R. J. (2005). Examination of fungal stress response genes using *Saccharomyces cerevisiae* as a model system: targeting genes affecting aflatoxin biosynthesis by *Aspergillus flavus* Link. *Appl. Microbiol. Biotechnol.* 67 807–815. 10.1007/s00253-004-1821-1 15614562

[B57] Kiran S. Kujur A. Prakash B. (2016). Assessment of preservative potential of *Cinnamomum zeylanicum* Blume essential oil against food borne molds, aflatoxin B1 synthesis, its functional properties and mode of action. *Innov. Food Sci. Emerg. Technol.* 37 184–191. 10.1016/j.ifset.2016.08.018

[B58] Kohiyama C. Y. Ribeiro M. M. Y. Mossini S. A. G. Bando E. da Silva Bomfim N. Nerilo S. B. (2015). Antifungal properties and inhibitory effects upon aflatoxin production of *Thymus vulgaris* L. by *Aspergillus flavus* Link. *Food Chem.* 173 1006–1010. 10.1016/j.foodchem.2014.10.135 25466118

[B59] Kumar A. Kujur A. Yadav A. Pratap S. Prakash B. (2019). Optimization and mechanistic investigations on antifungal and aflatoxin B1 inhibitory potential of nanoencapsulated plant-based bioactive compounds. *Ind. Crops Prod.* 131 213–223. 10.1016/j.indcrop.2019.01.043

[B60] Kumar P. Mahato D. K. Kamle M. Mohanta T. K. Kang S. G. (2017). Aflatoxins: a global concern for food safety, human health and their management. *Front. Microbial.* 7:2170 10.3389/fmicb.2016.02170 PMC5240007 28144235

[B61] Leslie J. F. Logrieco A. (2014). *Mycotoxin Reduction in Grain Chains.* Hoboken, NJ: Wiley Blackwell.

[B62] Liang D. Xing F. Selvaraj J. N. Liu X. Wang L. Hua H. (2015). Inhibitory effect of cinnamaldehyde, citral, and eugenol on aflatoxin biosynthetic gene expression and aflatoxin B1 biosynthesis in *Aspergillus flavus*. *J. Food Sci. Technol.* 80 M2917–M2924. 10.1111/1750-3841.13144 26556681

[B63] Liuzzi V. C. Fanelli F. Tristezza M. Haidukowski M. Picardi E. Manzari C. (2017). Transcriptional analysis of *Acinetobacter* sp. *neg1* capable of degrading ochratoxin A. *Front. Microbiol.* 7:2162. 10.3389/fmicb.2016.02162 28119679 PMC5220012

[B64] Loi M. Fanelli F. Cimmarusti M. T. Mirabelli V. Haidukowski M. Caliandro R. (2018). *In vitro* single and combined mycotoxins degradation by Ery4 laccase from *Pleurotus eryngii* and redox mediators. *Food Control* 90 401–406. 10.1016/j.foodcont.2018.02.032

[B65] Loi M. Fanelli F. Liuzzi V. C. Logrieco A. F. Mulè G. (2017). Mycotoxin biotransformation by native and commercial enzymes: present and future perspectives. *Toxins* 9:111. 10.3390/toxins9040111 28338601 PMC5408185

[B66] Loi M. Fanelli F. Zucca P. Liuzzi V. C. Quintieri L. Cimmarusti M. T. (2016). Aflatoxin B_1_ and M_1_ Degradation by Lac2 from *Pleurotus pulmonarius* and Redox Mediators. *Toxins* 8:245. 10.3390/toxins8090245 27563923 PMC5037472

[B67] Lopes L. F. Bordin K. de Lara G. H. Saladino F. Quiles J. M. Meca G. (2018). Fumigation of Brazil nuts with allyl isothiocyanate to inhibit the growth of *Aspergillus parasiticus* and aflatoxin production. *J. Sci. Food Agric.* 98 792–798. 10.1002/jsfa.8527 28675475 PMC6585674

[B68] Lv C. Wang P. Ma L. Zheng M. Liu Y. Xing F. (2018). Large-scale comparative analysis of eugenol-induced/repressed genes expression in *Aspergillus flavus* using RNA-seq. *Front. Microbiol.* 9:1116. 10.3389/fmicb.2018.01116 29899734 PMC5988903

[B69] Lyagin I. Efremenko E. (2019). Enzymes for detoxification of various mycotoxins: origins and mechanisms of catalytic action. *Molecules* 24:2362. 10.3390/molecules24132362 31247992 PMC6651818

[B70] Mahato D. K. Lee K. E. Kamle M. Devi S. Dewangan K. Kumar P. (2019). Aflatoxins in food and feed: an overview on prevalence, detection and control strategies. *Front. Microbiol.* 10:2266. 10.3389/fmicb.2019.02266 31636616 PMC6787635

[B71] Mahuku G. Nzioki H. S. Mutegi C. Kanampiu F. Narrod C. Makumbi D. (2019). Pre-harvest management is a critical practice for minimizing aflatoxin contamination of maize. *Food Control* 96 219–226. 10.1016/j.foodcont.2018.08.032 30713368 PMC6251936

[B72] Manyes L. Luciano F. B. Mañes J. Meca G. (2015). In vitro antifungal activity of allyl isothiocyanate (AITC) against *Aspergillus parasiticus* and *Penicillium expansum* and evaluation of the AITC estimated daily intake. *Food Chem. Toxicol.* 83 293–299. 10.1016/j.fct.2015.06.011 26140951

[B73] Marei G. I. K. Rasoul M. A. A. Abdelgaleil S. A. (2012). Comparative antifungal activities and biochemical effects of monoterpenes on plant pathogenic fungi. *Pest Biochem. Physiol.* 103 56–61. 10.1016/j.pestbp.2012.03.004

[B74] Mateo E. M. Gómez J. V. Domínguez I. Gimeno-Adelantado J. V. Mateo-Castro R. Gavara R. (2017). Impact of bioactive packaging systems based on EVOH films and essential oils in the control of aflatoxigenic fungi and aflatoxin production in maize. *Int. J. Food Microbiol.* 254 36–46. 10.1016/j.ijfoodmicro.2017.05.007 28525761

[B75] Miguel M. G. (2010). Antioxidant and anti-inflammatory activities of essential oils: a short review. *Molecules* 15 9252–9287. 10.3390/molecules15129252 21160452 PMC6259136

[B76] Mithöfer A. Maffei M. E. (2017). General mechanisms of plant defense and plant toxins. *Plant Toxins* 3–24. 10.1007/978-94-007-6728-7_21-1

[B77] Moon Y. S. Lee H. S. Lee S. E. (2018). Inhibitory effects of three monoterpenes from ginger essential oil on growth and aflatoxin production of *Aspergillus flavus* and their gene regulation in aflatoxin biosynthesis. *Appl. Biol. Chem.* 61 243–250. 10.1007/s13765-018-0352-x

[B78] Moretti A. Pascale M. Logrieco A. F. (2019). Mycotoxin risks under a climate change scenario in Europe. *Trends Food Sci. Technol.* 84 38–40. 10.1016/j.tifs.2018.03.008

[B79] Moretti A. Susca A. (2017). “Mycotoxigenic fungi: methods and protocols,” in *Methods in Enzimology*, Vol. vol. 1542 eds Moretti A. Susca A., (New York, NY: Humana Press), 10.1007/978-1-4939-6707-0

[B80] Nazareth T. M. Bordin K. Manyes L. Meca G. Mañes J. Luciano F. B. (2016). Gaseous allyl isothiocyanate to inhibit the production of aflatoxins, beauvericin and enniatins by *Aspergillus parasiticus* and *Fusarium poae* in wheat flour. *Food Control* 62 317–321. 10.1016/j.foodcont.2015.11.003

[B81] Negera M. Washe A. P. (2019). Use of natural dietary spices for reclamation of food quality impairment by aflatoxin. *J. Food Qual.* 2019:4371206 10.1155/2019/4371206

[B82] Neme K. Mohammed A. (2017). Mycotoxin occurrence in grains and the role of postharvest management as a mitigation strategies. A review. *Food Control* 78 412–425. 10.1016/j.foodcont.2017.03.012

[B83] Omidpanah S. Sadeghi H. Sarcheshmeh M. M. Manayi A. (2015). Evaluation of antifungal activity of aqueous extracts of some medicinal plants against *Aspergillus flavus*, pistachio aflatoxin producing fungus *in vitro*. *Drug Des. Dev. Ther.* 6 66–69. 10.4103/2394-6555.162446

[B84] Onaran A. Yanar Y. (2016). *In vivo* and *in vitro* antifungal activities of five plant extracts against various plant pathogens. *Egypt. J. Biol. Pest Control* 26 405. 10.1016/j.pestbp.2018.07.001 30195388

[B85] OuYang Q. Tao N. Jing G. (2016). Transcriptional profiling analysis of *Penicillium digitatum*, the causal agent of citrus green mold, unravels an inhibited ergosterol biosynthesis pathway in response to citral. *BMC Genomics* 17:599. 10.1186/s12864-016-2943-4 27514516 PMC4982135

[B86] Panda P. Mehta A. (2013). Aflatoxin detoxification potential of *Ocimum Tenuiflorum*. *J. Food Saf.* 33 265–272. 10.1111/jfs.12048

[B87] Peretto G. Du W. X. Avena-Bustillos R. J. Sarreal S. B. L. Hua S. S. T. Sambo P. (2014). Increasing strawberry shelf-life with carvacrol and methyl cinnamate antimicrobial vapors released from edible films. *Postharvest Biol. Technol.* 89 11–18. 10.1016/j.postharvbio.2013.11.003

[B88] Pichersky E. Raguso R. A. (2018). Why do plants produce so many terpenoid compounds? *New Phytol.* 220 692–702. 10.1111/nph.14178 27604856

[B89] Pinto E. Hrimpeng K. Lopes G. Vaz S. Gonçalves M. J. Cavaleiro C. (2013). Antifungal activity of *Ferulago capillaris* essential oil against Candida, Cryptococcus, Aspergillus and dermatophyte species. *Eur. J Clin. Microbiol. Infect Dis.* 32 1311–1320. 10.1007/s10096-013-1881-1 23619574

[B90] Pisoschi A. M. Pop A. Cimpeanu C. Predoi G. (2016). Antioxidant capacity determination in plants and plant-derived products: a review. *Oxid. Med. Cell. Longev.* 2016:9130976. 10.1155/2016/9130976 28044094 PMC5164913

[B91] Ponzilacqua B. Rottinghaus G. E. Landers B. R. Oliveira C. A. F. (2019). Effects of medicinal herb and Brazilian traditional plant extracts on *in vitro* mycotoxin decontamination. *Food Control* 100 24–27. 10.1016/j.foodcont.2019.01.009

[B92] Prakash B. Kedia A. Mishra P. K. Dubey N. K. (2015). Plant essential oils as food preservatives to control moulds, mycotoxin contamination and oxidative deterioration of agri-food commodities–Potentials and challenges. *Food Control* 47 381–391. 10.1016/j.foodcont.2014.07.023

[B93] Quiles J. M. Manyes L. Luciano F. Manes J. Meca G. (2015). Influence of the antimicrobial compound allyl isothiocyanate against the *Aspergillus parasiticus* growth and its aflatoxins production in pizza crust. *Food Chem. Toxicol.* 83 222–228. 10.1016/j.fct.2015.06.017 26146190

[B94] Quiles J. M. Nazareth T. D. M. Luz C. Luciano F. B. Mañes J. Meca G. (2019). Development of an Antifungal and Antimycotoxigenic Device Containing Allyl Isothiocyanate for Silo Fumigation. *Toxins* 11:E137. 10.3390/toxins11030137 30823642 PMC6468390

[B95] Rammanee K. Hongpattarakere T. (2011). Effects of tropical citrus essential oils on growth, aflatoxin production, and ultrastructure alterations of *Aspergillus flavus* and *Aspergillus parasiticus*. *Food Bioprocess Technol.* 4 1050–1059. 10.1007/s11947-010-0507-1

[B96] Rao A. Zhang Y. Muend S. Rao R. (2010). Mechanism of antifungal activity of terpenoid phenols resembles calcium stress and inhibition of the TOR pathway. *Antimicrob. Agents Chemother.* 54 5062–5069. 10.1128/AAC.01050-10 20921304 PMC2981246

[B97] Regulation (EC) No 1831/2003 (2003). *European Parliament, and of the Council of 22 September 2003 on Additives for Use in Animal Nutrition (OJ L 268, 18.10.2003).* Available at: https://eur-lex.europa.eu/legal-content/EN/LSU/?uri=CELEX:02003R1831-20151230, 29–43 (accessed September 12, 2019).

[B98] Reverberi M. Fabbri A. A. Zjalic S. Ricelli A. Punelli F. Fanelli C. (2005). Antioxidant enzymes stimulation in *Aspergillus parasiticus* by *Lentinula edodes* inhibits aflatoxin production. *Appl. Microbiol. Biotechnol.* 69 207–215. 10.1007/s00253-005-1979-1 15838675

[B99] Reverberi M. Ricelli A. Zjalic S. Fabbri A. A. Fanelli C. (2010). Natural functions of mycotoxins and control of their biosynthesis in fungi. *Appl. Microbiol. Biotechnol.* 87 899–911. 10.1007/s00253-010-2657-5 20495914

[B100] Reverberi M. Zjalic S. Ricelli A. Fabbri A. A. Fanelli C. (2006). Oxidant/antioxidant balance in *Aspergillus parasiticus* affects aflatoxin biosynthesis. *Mycotoxin Res.* 22 39–47. 10.1007/BF02954556 23605500

[B101] Saladino F. Bordin K. Manyes L. Luciano F. B. Manes J. Fernández-Franzón M. (2016). Reduction of the aflatoxins B1, B2, G1 and G2 in Italian piadina by isothiocyanates. *LWT Food Sci. Technol.* 70 302–308. 10.1016/j.lwt.2016.03.006

[B102] Sant D. G. Tupe S. G. Ramana C. V. Deshpande M. V. (2016). Fungal cell membrane-promising drug target for antifungal therapy. *J. Appl. Microbiol.* 121 1498–1510. 10.1111/jam.13301 27667746

[B103] Sharma M. Koul A. Sharma D. Kaul S. Swamy M. K. Dhar M. K. (2019). “Metabolic engineering strategies for enhancing the production of bio-active compounds from medicinal plants,” in *Natural Bio-active Compounds*, eds Akhtar M. Swamy M., (Singapore: Springer), 10.1007/978-981-13-7438-8_12

[B104] Sharma N. Tripathi A. (2008). Effects of *Citrus sinensis* (L.) Osbeck epicarp essential oil on growth and morphogenesis of *Aspergillus niger* (L.) Van Tieghem. *Microbiol. Res.* 163 337–344. 10.1016/j.micres.2006.06.009 16870411

[B105] Shen Q. Zhou W. Li H. Hu L. Mo H. (2016). ROS involves the fungicidal actions of thymol against spores of *Aspergillus flavus* via the induction of nitric oxide. *PLoS One* 11:e0155647. 10.1371/journal.pone.0155647 27196096 PMC4872997

[B106] Sivakumar D. Bautista-Baños S. (2014). A review on the use of essential oils for postharvest decay control and maintenance of fruit quality during storage. *Crop Prot.* 64 27–37. 10.1016/j.cropro.2014.05.012

[B107] Steiner T. Syed B. (2015). “Phytogenic feed additives in animal nutrition,” in *Medicinal and Aromatic Plants of the World*, ed. Máthé Á., (Dordrecht: Springer), 403–423. 10.1007/978-94-017-9810-5_20

[B108] Stevanović Z. Bošnjak-Neumüller J. Pajić-Lijaković I. Raj J. Vasiljević M. (2018). Essential oils as feed additives—future perspectives. *Molecules* 23:1717. 10.3390/molecules23071717 30011894 PMC6100314

[B109] Sudharsan S. Shapiro O. Ziv C. Barda O. Zakin V. Sionov E. (2019). Synergistic Inhibition of Mycotoxigenic Fungi and Mycotoxin Production by Combination of Pomegranate Peel Extract and Azole Fungicide. *Front. Microbiol.* 10:1919. 10.3389/fmicb.2019.01919 31481948 PMC6710344

[B110] Sun Q. Shang B. Wang L. Lu Z. Liu Y. (2016). Cinnamaldehyde inhibits fungal growth and aflatoxin B 1 biosynthesis by modulating the oxidative stress response of *Aspergillus flavus*. *Appl. Microbiol. Biot.* 100 1355–1364. 10.1007/s00253-015-7159-z 26585445

[B111] Tabassum N. Vidyasagar G. M. (2013). Antifungal investigations on plant essential oils. A review. *Int. J. Pharm. Pharm. Sci.* 5 19–28.

[B112] Tian J. Ban X. Zeng H. He J. Chen Y. Wang Y. (2012a). The mechanism of antifungal action of essential oil from dill (*Anethum graveolens* L.) *on Aspergillus flavus*. *PLoS One* 7:e30147. 10.1371/journal.pone.0030147 22272289 PMC3260232

[B113] Tian J. Ban X. Zeng H. He J. Huang B. Wang Y. (2011). Chemical composition and antifungal activity of essential oil from *Cicuta virosa* L. *var. latisecta Celak. ý* *Int. J. Food Microbiol.* 145 464–470. 10.1016/j.ijfoodmicro.2011.01.023 21320730

[B114] Tian J. Huang B. Luo X. Zeng H. Ban X. He J. (2012b). The control of *Aspergillus flavus* with *Cinnamomum jensenianum* Hand.-Mazz essential oil and its potential use as a food preservative. *Food Chem.* 130 520–527. 10.1016/j.foodchem.2011.07.061

[B115] Tiihonen K. Kettunen H. Bento M. H. L. Saarinen M. Lahtinen S. Ouwehand A. C. (2010). The effect of feeding essential oils on broiler performance and gut microbiota. *Br. Poult. Sci.* 51 381–392. 10.1080/00071668.2010.496446 20680873

[B116] Tracz B. L. Bordin K. de Melo Nazareth T. Costa L. B. de Macedo R. E. F. Meca G. (2017). Assessment of allyl isothiocyanate as a fumigant to avoid mycotoxin production during corn storage. *LWT Food Sci. Technol.* 75 692–696. 10.1016/j.lwt.2016.10.030

[B117] Upadhyay S. Dixit M. (2015). Role of polyphenols and other phytochemicals on molecular signaling. *Oxid. Med. Cell. Longev.* 2015:504253. 10.1155/2015/504253 26180591 PMC4477245

[B118] Velazhahan R. Vijayanandraj S. Vijayasamundeeswari A. Paranidharan V. Samiyappan R. Iwamoto T. (2010). Detoxification of aflatoxins by seed extracts of the medicinal plant, *Trachyspermum ammi* (L.) Sprague ex Turrill–structural analysis and biological toxicity of degradation product of aflatoxin G1. *Food Control* 21 719–725. 10.1016/j.foodcont.2009.10.014 10887046

[B119] Vijayanandraj S. Brinda R. Kannan K. Adhithya R. Vinothini S. Senthil K. (2014). Detoxification of aflatoxin B1 by an aqueous extract from leaves of *Adhatoda vasica* Nees. *Microbiol. Res.* 169 294–300. 10.1016/j.micres.2013.07.008 23928380

[B120] Walters D. (2011). *Plant Defense: Warding off Attack by Pathogens, Herbivores and Parasitic Plants.* Hoboken, NJ: Blackwell Publishing 10.1002/9781444328547

[B121] Wu Y. Luo Y. Wang Q. (2012). Antioxidant and antimicrobial properties of essential oils encapsulated in zein nanoparticles prepared by liquid–liquid dispersion method. *LWT Food Sci. Technol.* 48 283–290. 10.1016/j.lwt.2012.03.027

[B122] Xie X. M. Fang J. R. Xu Y. (2004). Study of antifungal effect of cinnamaldehyde and citral on *Aspergillus flavus*. *Food Sci.* 25 32–34.

[B123] Xie Y. Huang Q. Wang Z. Cao H. Zhang D. (2017). Structure-activity relationships of cinnamaldehyde and eugenol derivatives against plant pathogenic fungi. *Ind. Crops Prod.* 97 388–394. 10.1016/j.indcrop.2016.12.043

[B124] Yu J. Chang P. K. Ehrlich K. C. Cary J. W. Bhatnagar D. Cleveland T. E. (2004). Clustered pathway genes in aflatoxin biosynthesis. *Appl. Environ. Microbiol.* 70 1253–1262. 10.1128/AEM.70.3.1253-1262.2004 15006741 PMC368384

[B125] Zeng Z. Zhang S. Wang H. Piao X. (2015). Essential oil and aromatic plants as feed additives in non-ruminant nutrition: a review. *J. Anim. Sci. Biotechnol.* 6:7. 10.1186/s40104-015-0004-5 25774291 PMC4359495

[B126] Zhang H. Tsao R. (2016). Dietary polyphenols, oxidative stress and antioxidant and anti-inflammatory effects. *Curr. Opin. Food Sci.* 8 33–42. 10.1016/j.cofs.2016.02.002

